# Systemic corticosteroids improve tendon healing when given after the early inflammatory phase

**DOI:** 10.1038/s41598-017-12657-0

**Published:** 2017-09-29

**Authors:** Parmis Blomgran, Malin Hammerman, Per Aspenberg

**Affiliations:** Department of Clinical and Experimental Medicine, Linköping University, Linköping, Sweden

## Abstract

Inflammation initiates tendon healing and then normally resolves more or less completely. Unresolved inflammation might disturb the remodeling process. We hypothesized that suppression of inflammation during the early remodeling phase by systemic dexamethasone treatment can improve healing. 36 rats underwent Achilles tendon transection and were randomized to dexamethasone or saline on days 0–4 after surgery (early inflammatory phase), and euthanasia day 7. Another 54 rats received injections days 5–9 (early remodeling phase) and were euthanized day 12 for mechanical, histological and flow cytometric evaluation. Dexamethasone treatment days 0–4 reduced the cross-sectional area, peak force and stiffness by day 7 to less than half (p < 0.001 for all), while material properties (peak stress and elastic modulus) were not significantly affected. In contrast, dexamethasone treatment days 5–9 *increased* peak force by 39% (p = 0.002) and stiffness by 58% (p < 0.001). The cross-sectional area was reduced by 42% (p < 0.001). Peak stress and elastic modulus were more than doubled (p < 0.001 for both). Semi-quantitative histology at day 12 showed that late dexamethasone treatment improved collagen alignment, and flow cytometry revealed reduced numbers of CD8a^+^ cytotoxic T cells in the tendon callus. These results suggest that downregulation of lingering inflammation during the early remodeling phase can improve healing.

## Introduction

Tendon healing starts with an inflammatory phase that soon resolves more or less completely. Both the inflammation and its resolution are thought to be required for optimal healing, suggesting that a prolonged inflammation would have negative effects for the formation of new tendon-like tissue. Inhibition of inflammation with non-steroidal anti-inflammatory drugs during the early inflammatory phase has a detrimental effect on tendon healing^1,2^. In contrast, they have a slight positive effect if given later during the remodeling phase^1^. Thus, inflammation might play different roles during different phases of healing, and anti-inflammatory drugs might have different effects depending on when they are given. Already in 1950, it was shown that systemic glucocorticoid treatment reduced skin wound strength^3^. Wound shrinking was also reduced by early treatment, but if treatment started first on day 3, the effect was absent^4^. Later research has mainly focused on local treatment, reporting deleterious effect of locally injected corticosteroids on intact and healing tendons^5–9^, due to anti-inflammatory effects and suppressed collagen synthesis and fibroblast proliferation^10,11^. Although most studies show a detrimental effect of local corticosteroid injection on intact tendons^12–14^ also a positive effect has been described^15^.

Systemic treatment, however, might have quite different effects from local. Most of the cells in the early tendon callus in a rat Achilles tendon transection model are leukocytes^16^. Because regeneration in this model starts with an empty tendon defect, most of these cells are likely to be recruited to the site from the bone marrow. The cells that later on down-regulate the inflammation, like alternatively activated macrophages and regulatory T cells, are also likely to derive from the bone marrow. Therefore, systemic glucocorticoids that act on the immune system as a whole, including the bone marrow, might have effects that are different from local treatment. There seems to be no data on *systemic* glucocorticoid treatment and tendon healing. There is, however, some data on fracture healing, showing a detrimental effect on shaft fracture healing of systemic glucocorticoids, but a slight positive effect on cancellous bone healing^17^.

In a rat Achilles tendon healing model, there is a considerable proportion of leukocytes in the regenerating tissue still at 10 days after tendon transection, a time point where the tendon has regained almost half of its strength^16^. This suggests that inflammation is still ongoing during the early remodeling phase of tendon healing. We hypothesized that inflammation at this time might interfere with remodeling, and that further reduction of this lingering inflammation with systemic corticosteroids would improve healing.

## Results

### Mechanics

**Figure 1:**
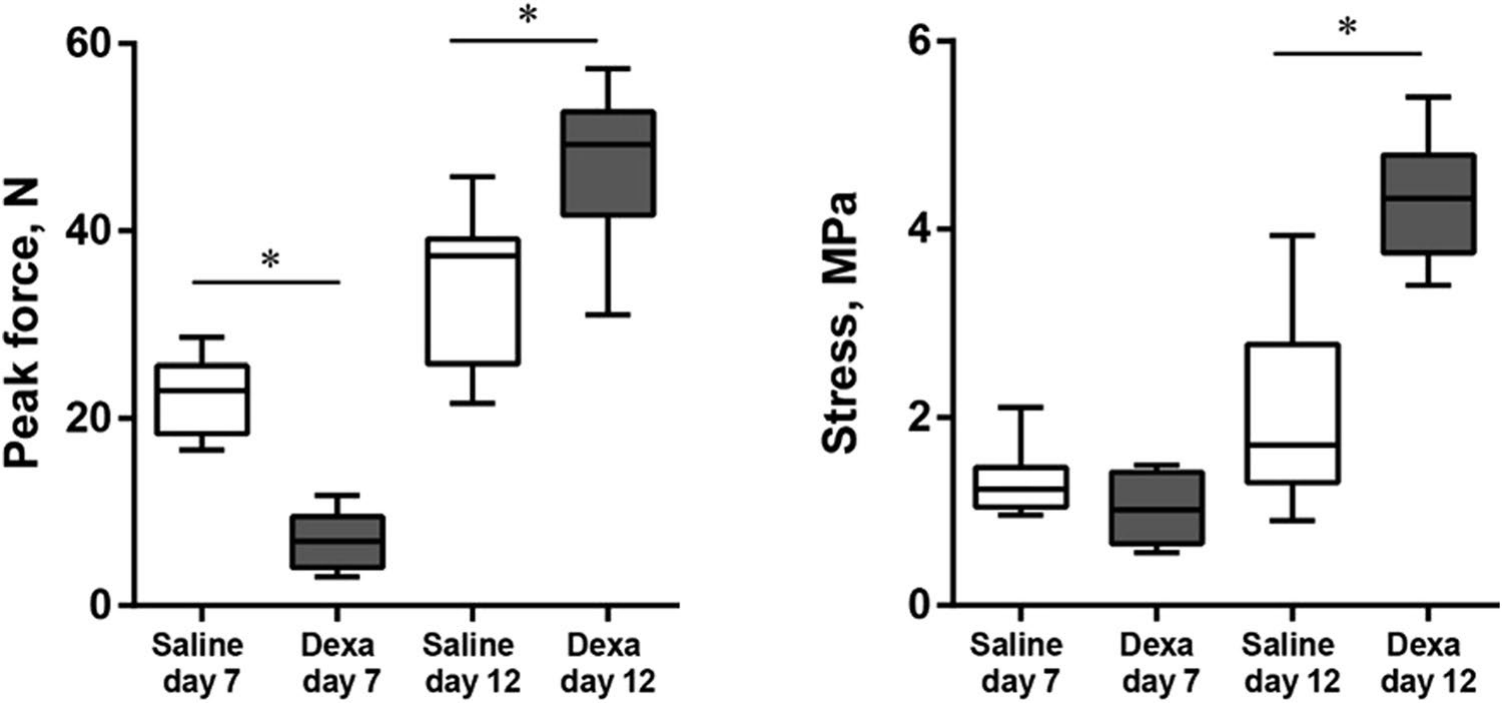
Peak force and stress at destructive tensional testing 7 and 12 days after tendon transection. For testing day 7, animals received dexamethasone or saline days 0–4, and for testing day 12, the treatment was given days 5–9. N = 10 or 11 rats in each group.

We first transected the right Achilles tendon on 24 rats, and randomized them to either systemic dexamethasone treatment or saline during the inflammatory phase of healing (days 0-4). Destructive tensional testing of the healing tendon Day 7 showed that dexamethasone reduced the peak force, stiffness and cross-sectional area of the healing tissue to less than half compared with saline at the same day (p < 0.001 for all). Material properties seemed unaffected: peak stress and modulus of elasticity were similar (Fig. 1, Table 1). Next, we came to the main experiment: 24 rats were randomized to systemic dexamethasone or saline during the early remodeling phase (days 5–9) and killed on Day 12. Peak force was the primary effect variable. With this timing, dexamethasone increased peak force by 39 percent (p = 0.002; 95% CI 17 to 62 percent), stiffness by 58 percent, while still reducing the cross-sectional area by 42 percent. Material properties were improved: peak stress and modulus of elasticity were both more than doubled (p<0.001 for all mentioned variables, Fig. 1, Table 1).

**Table 1:** Mechanical properties of rat Achilles tendons at 7 days and 12 days after tendon transection.

			Dexa (SD)	p-value
Day 7	Peak force, N	22.2 (4.1)	7.0 (3.0)	0.000
	Stiffness, N/mm	3.2 (0.5)	1.1 (0.6)	0.000
	Peak stress MPa	1.3 (0.3)	1.0 (0.4)	0.089
	Cross sectional area, mm^2^	17.4 (3.9)	6.5 (1.8)	0.000
	Elastic Modulus (MPa)	2.7 (0.6)	2.6 (1.3)	0.8
	Energy (Nmm)	48.4 (11.2)	11.4 (6.9)	0.000
Day 12	Peak force, N	33.6 (8.2)	46.8 (8.3)	0.002
	Stiffness, N/mm	4.7 (0.9)	7.4 (1.6)	0.000
	Peak stress MPa	1.9 (0.9)	4.3 (0.7)	0.000
	Cross sectional area, mm^2^	18.8 (4.9)	10.9 (2.5)	0.000
	Elastic Modulus (MPa)	3.6 (1.6)	9.3 (2.5)	0.000
	Energy (Nmm)	84.3 (21.5)	101 (22.5)	0.1

.

### Histology

**Figure 2:**
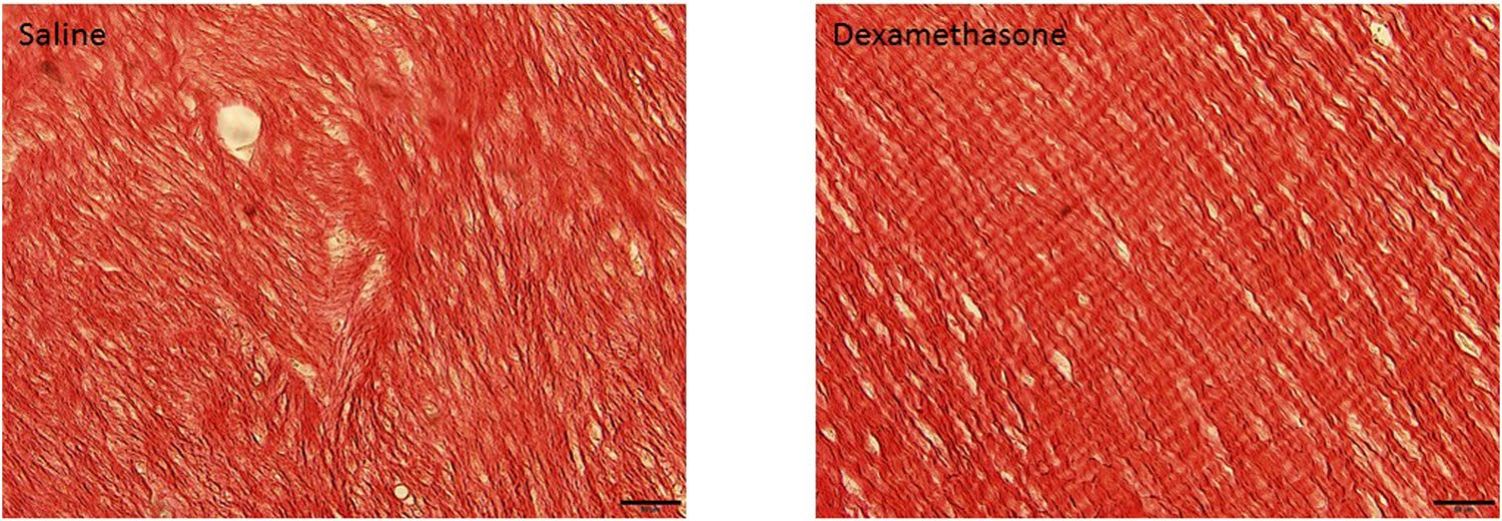
Representable samples with picrosirius staining. Note a higher degree of collagen organization with dexamethasone. Bar length 50 µm.

The observation that late dexamethasone treatment increased the peak force in spite of a reduced cross-sectional area suggested a better collagen organization. We therefore performed a microscopic evaluation of 18 specimens treated days 5–9 and euthanized Day 12, using an arbitrary score for degree of tissue organization (Fig. 2). A blinded scoring by an experienced microscopist (PA) showed a higher degree of tissue organization in the dexamethasone samples compared to saline (P =  0.007; Table 2). A repeated scoring by the same person 16 weeks later revealed an identical result for all specimens except one (Intrarater correlation by Spearman’s rho 0.93; Table 2). An less experienced microscopist (MH) was also able to distinguish the treatments groups, but with less certainty (p = 0.039; interrater correlation 0.68).

**Table 2:** Blinded histological grading of collagen organization as low, middle and high. An experienced reviewer graded the specimens twice with 16 weeks in between.

Reviewer	Evaluation	Sample	Low	Middle	High
Experienced	First	Saline	5	3	0
		Dexamethasone	0	5	3
Experienced	Second	Saline	6	2	0
		Dexamethasone	0	5	3
Less experienced	First	Saline	4	3	1
		Dexamethasone	1	2	5

Dexamethasone samples showed a higher degree of tissue organization compared to saline (P = 0.007). As a control, a less experienced reviewer then graded the specimens and confirmed a difference between dexamethasone and saline (p = 0.039).

### Flow cytometry

**Figure 3:**
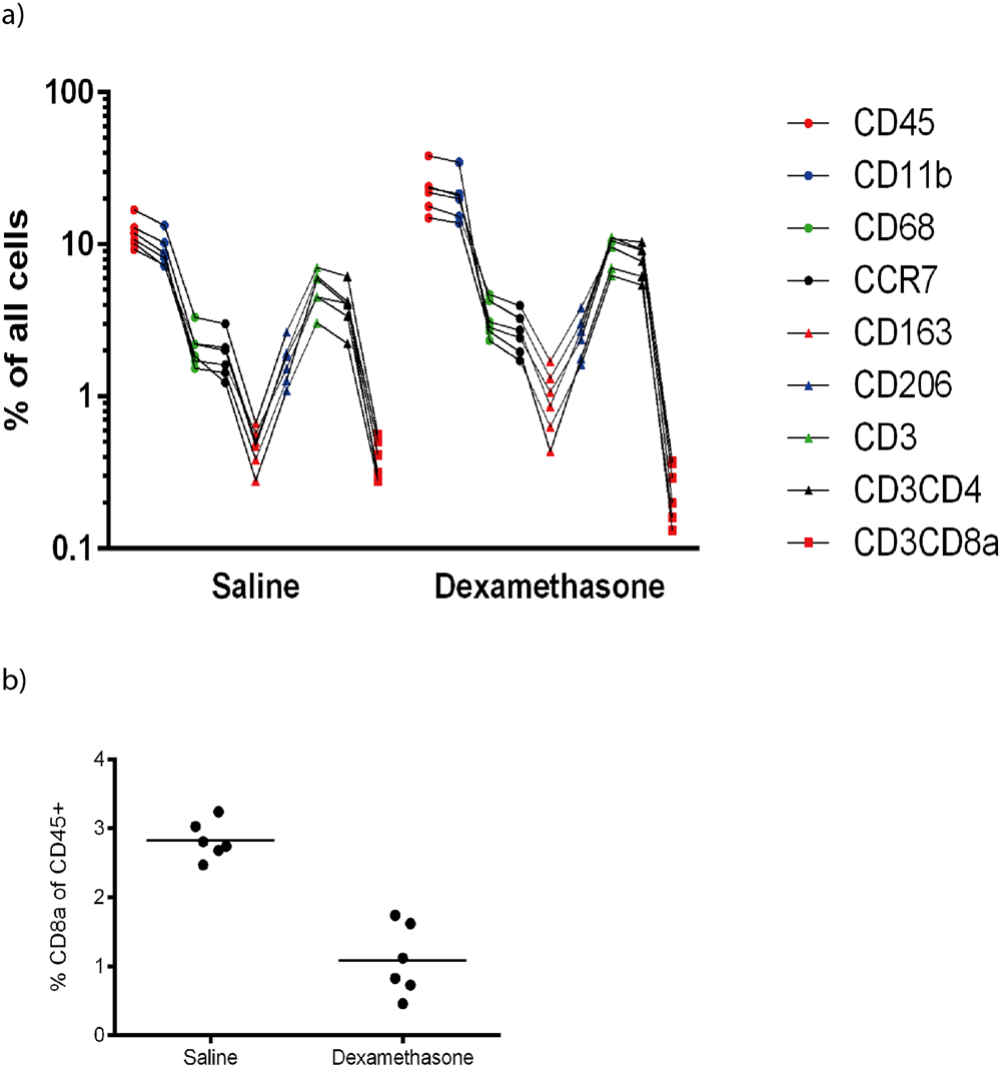
(**a**) Number of cells labelled with markers as a percent of all cells, 7 days after tendon transection. Each measurement is shown by a point, and all measurements from the same rat are connected by a line, to visualize an “inflammatory signature”. Markers are aligned in the following order: CD45 (leukocytes), CD11b (phagocytes), CD68 (pan-macrophages), CCR7, CD163, CD206 (macrophage subtypes), CD3, CD4, CD8a (T cell and T cell subtypes). N = 6 rats in each group. Note lower CD8a in the dexamethasone group. (**b**) Cytotoxic (CD3^+^CD8a^+^) T cells in the tendon healing tissue 7 days after surgery. The cells are presented as percent of all leukocytes (CD45^+^).

**Figure 4:**
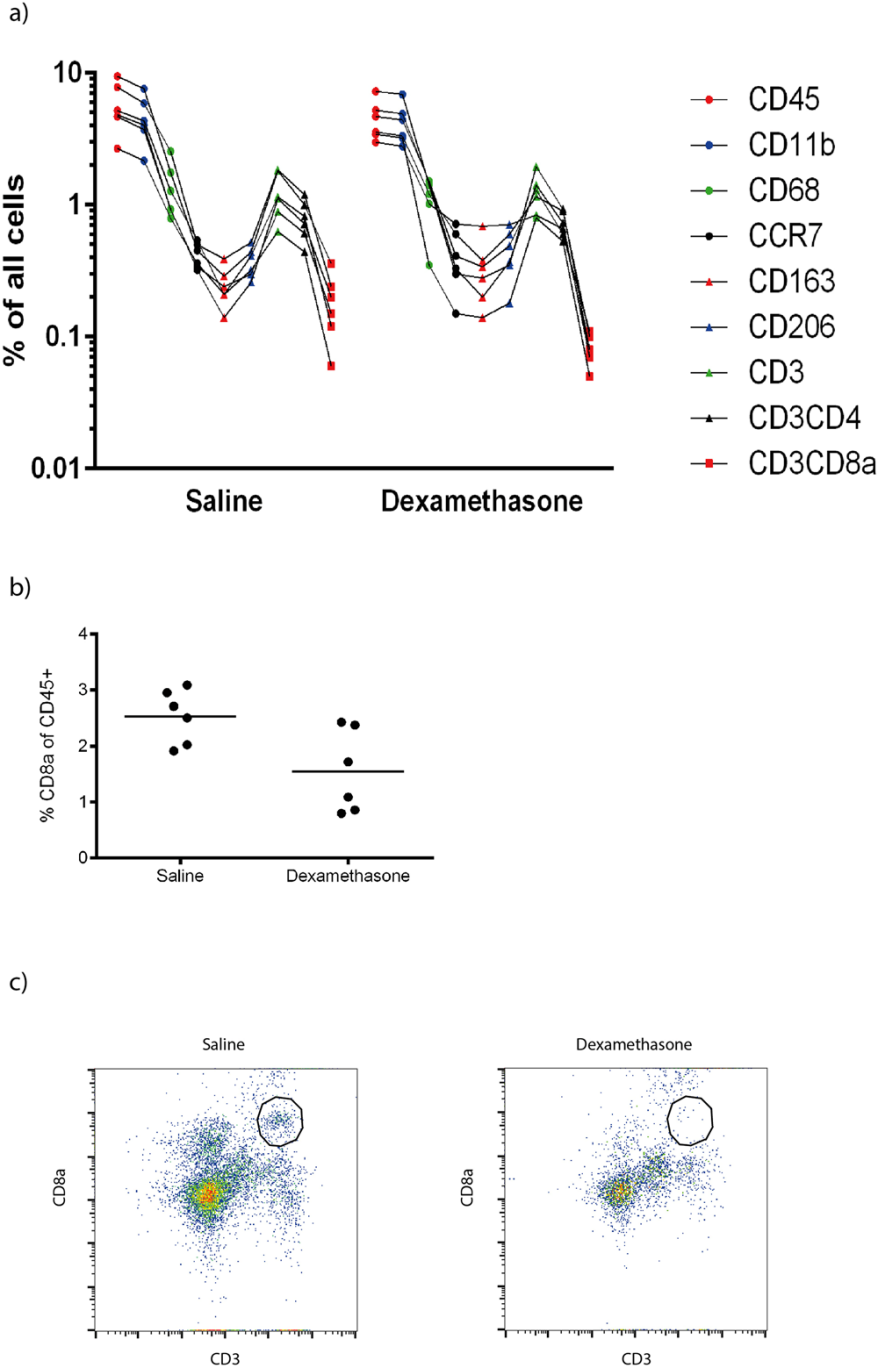
(**a**) Number of cells labelled with markers as a percent of all cells, 12 days after tendon transection. Each measurement is shown by a point, and all measurements from the same rat are connected by a line, to visualize an “inflammatory signature”. Markers are aligned in the following order: CD45 (leukocytes), CD11b (phagocytes), CD68 (pan-macrophages), CCR7, CD163, CD206 (macrophage subtypes), CD3, CD4, CD8a (T cell and T cell subtypes). N = 6 in each group. Note greater variation in the dexamethasone group, and lower CD8a. (**b**) Cytotoxic (CD3^+^CD8a^+^) T cells in the tendon healing tissue 12 days after surgery. The cells are presented as percent of all leukocytes (CD45^+^). The CD8a cytotoxic T cell subpopulation was reduced in dexamethasone samples (p = 0.03). (**c**) Flow cytometry plots showing a distinct CD3^+^CD8a^+^ population in the saline group and no such distinct cell population in the dexamethasone treated group.See supplementary Figure S1 for plots from all animals.

To determine if the effect of dexamethasone was also related to changes in the immune cell populations within the healing tendons, we performed a flow cytometric analysis on samples from rats treated days 0–4 and 5–9 and killed on day 7 and 12 respectively. On Day 7 the proportion of leukocytes (CD45^+^) was increased in the dexamethasone treated specimens, suggesting that proliferation of other (mesenchymal) cells was reduced. However, the CD8a cytotoxic T cell subpopulation was drastically reduced (Fig. 3a,b). On day 12, no statistically significant differences in cell proportions were found, except, again, for a clear reduction of CD8a cytotoxic T cell subpopulation in all dexamethasone samples (Mann-Whitney test, p = 0.03). The flow cytometric cloud of these cells was absent (Fig. 4a,b,c).

## Discussion

We here show that dexamethasone treatment, initiated after the early inflammatory phase, improves tendon healing. To our knowledge, similar effects on healing of collagenous tissues have not been previously reported.

The doubling in peak stress corresponded to an improvement in tissue organization that was so obvious that it could be confirmed with semi-quantitative histology. It seems reasonable to assume that inflammation that carries on after the initial phase of healing disturbs the organization of the collagenous tissue being formed. The numbers of most leukocyte subpopulations studied in the tissue 12 days after surgery were not much changed by dexamethasone treatment, with exception for the CD8a cytotoxic T cells.

This cell subpopulation has been implicated in healing before. High levels of certain CD8 T cell fractions in the circulation are associated with poor fracture healing in humans^18^. In mice, deletion of CD8 T cells improves fracture healing and infusion of such cells impairs it, and the fact that a wild-type microbiota impairs fracture healing in laboratory mice seems to be explained by increasing CD8 T cell numbers^18^. These cells are known to play multiple roles: even though they belong to the adaptive immune system, they can be activated without antigens and exert both cytotoxic and secretory functions, such as producing large amounts of interferon gamma^18^. Our findings regarding CD8a T cells for late tendon healing and tissue organization are similar to the above mentioned fracture findings, where a causal relation appeared likely. We are unable to explain why low CD8a T cell counts are associated with poor mesenchymal cell proliferation in early healing, but the negative effect of dexamethasone during this phase is well known, and might be exerted via other mechanisms overriding any role CD8a T cell changes. Moreover, we have recently found in the same tendon healing model as in the current study that the microbiome, and thereby the immune status of the rats, influences the tendon healing response to locally injected platelets^19^.

Our findings might be unrelated to corticosteroid injections given locally. Immune cells in the tendon callus have arrived from the bone marrow, and as we gave the dexamethasone systemically, its effects might have been exerted in the marrow. A difference from local injections might be supported by the observation that delayed local injections in torn rabbit collateral ligaments showed only detrimental effects^20^. Still, it is theoretically impossible to claim that a positive effect of any form of local injections can be excluded.

Even though corticosteroids are often thought of as general down regulators of inflammation, we saw no reduction in the proportion of leukocytes in the healing tissue at any time point. We were surprised by this finding, but consider further analysis of the underlying mechanism beyond the scope of this investigation. It seems the number of cells might be less important than what signals they release, something we have not studied. Corticosteroids have been implicated to impair healing by reducing e.g. TGF-beta expression, but also via direct effects on mesenchymal cells reducing their proliferation^21,22^. Still against the background of the fracture healing literature, the dramatic reduction in CD8a cytotoxic T cells seems to play a crucial role.

This study has consistently used randomization and blinded evaluation. Other strengths include the use of animals in an old animal facility, where they were exposed to a bacterially rather contaminated environment, which is likely to increase CD8a levels. Animals not challenged by pathogens would have lower levels of CD8a T cells, and might not respond in the same way to dexamethasone. Laboratory animals with an unchallenged immune system are suggested to be irrelevant for humans^23^.

It is of course a stretch to extrapolate the findings from this study to treatment of tendon ruptures in humans. Still, it seems appropriate to give more thought to the resolution of inflammation after various orthopedic injuries. It is tempting to speculate that systemic corticosteroids, given from e.g. day 10 and onwards in humans, might accelerate the healing process.

Another limitation of this work is that the severed Achilles tendons were healthy before the injury, while human ruptured tendons may be affected by tendinopathy. Studies with biopsies from humans with supraspinatus ruptures show inflammatory changes also in the intact subscapularis tendon both on the cellular and cytokine levels^24,25^. It seems clear that tendinopathy is associated with an increased presence of macrophages, mast cells or T cells^26^ . However, because tendon healing after rupture mainly means formation of new tissue in the form of a tendon callus, pre-existing inflammation in the ruptured stumps is probably unlikely to play a crucial role.

In conclusion, dexamethasone, given from day 5 onwards after Achilles tendon transection, improved tissue strength and quality. This effect was possibly due to a reduction in the number of cytotoxic T cells.

## Materials and Methods

### Study design

We used 90 female Sprague-Dawley rats (11–12 weeks old); 48 animals for mechanical evaluation, 18 for histology and 24 for flow cytometry. The right Achilles tendon was transected in all rats, and allowed to heal spontaneously without suture. Rats were randomized to dexamethasone treatment or saline control. The day of surgery was regarded as day 0.

To evaluate the mechanical properties of the healing tendon, rats received a daily dose of dexamethasone or saline day 0–4 and were euthanized day 7, or received a daily dose of dexamethasone or saline day 5–9 and were euthanized day 12. Healing tendons were evaluated by mechanical testing (n = 12 in each group).

Histological (n = 9 in each group) and flow cytometric (n = 6 in each group) evaluation were performed to further illuminate the mechanical data. For this, the rats were randomized to a daily dose of dexamethasone or saline given days 5–9 and were killed day 12 for histology evaluation or received dexamethasone or saline given days 0–4 and 5–9 and were killed day 7 and 12 for flow cytometry evaluation.

All experiments were approved by the Regional Ethics Committee for animal experiments in Linköping and adhered to the institutional guidelines for care and treatment of laboratory animals. The rats were housed 2 or 3 per cage and given food and water ad libitum.

### Surgery

Rats were anesthetized with isoflurane gas (Forene, Abbot Scandinavia, Solna, Sweden) and received antibiotics (25 mg/kg, Oxytetracycline, Engemycin; Intervet, Boxmeer, The Netherlands) preoperatively and analgesics (0.045 mg/kg, Buprenorphine, Temgesic; Schering-Plough, Brussels, Belgium) was given subcutaneously pre and postoperatively. The skin on the right Achilles tendon was shaved and cleaned with chlorhexidine ethanol. Thereafter, a transverse skin incision was made lateral to the Achilles tendon. The plantaris tendon was removed and the Achilles tendon was cut transversely and the tendon was left unsutured to heal spontaneously. The skin was sutured with two stitches.

### Drug administration

Dexamethasone was administered 0.5 mg/kg body weight subcutaneously once daily (Dexaject, Dopharma, Denmark). For evaluation on day 7, rats received dexamethasone days 0–4, and for evaluation on day 12, rats received dexamethasone days 5–9. Control animals received saline subcutaneously days 0–4 or 5–9.

### Mechanical evaluation

Rats were euthanized by CO_2_. The Achilles tendon with the calcaneal bone and gastrocnemius and soleus muscles was harvested. The muscle was scraped off and tendon fibers were fixed by sand paper in a metal clamp. The calcaneal bone was fixed in a custom-made clamp at 30° dorsiflexion relative to the direction of traction. The mechanical testing machine (100R, DDL, Eden Praire, MN) pulled the mounted tendon at constant speed (0.1 mm/s) until failure. Data acquisition rate was 1/0.03 s. Peak force at failure (N), stiffness (N/mm), and energy uptake (Nmm) were calculated by the testing machine. Investigator marked the linear part of the elastic phase in the force extension curve in order to calculate stiffness. Sagittal and transverse diameter of the mid part of the callus were measured by a caliper. Cross-sectional area, Young’s modulus and peak stress were calculated assuming an elliptic cylindrical shape as described previously^2^. All measurements were performed by a blinded investigator.

### Tissue harvest and retrieval of single cells

Rats were euthanized by CO_2_ and the tendons retrieved. To ensure excision of only newly formed healing tissue between the resection ends, two cuts were made, 4 mm apart, perpendicular to the direction of the tendon in the middle of the former defect. The excised specimens were placed in digestion buffer (RPMI 1640 with, 5% heat inactivated fetal bovine serum, and 10 mM HEPES). The specimens were minced into small pieces and incubated with 1 mg/mL Collagenase D (Roche) and 30 µg/ml DNase (Roche) at 37 °C for 45 min. Specimens were passed through a 70 μm cell strainer (Fisher scientific). Single cells were washed, and RBC was removed using ACK lysis buffer (155 mM NH3Cl, 10 mM KHCO3, and 88 µM EDTA). Trypan blue (Life technologies) was used to count live cells.

### Flow cytometric phenotyping of immune cells

Antibodies were CD45-PE-Cy7 (leukocyte), CD3-AF647 (T cell), CD4-PE (T helper cell), CD25-BV510 and Foxp3-AF488 (regulatory T cell), CD8a-PerCP (cytotoxic T cell) from Biolegend and CD11b-AF700 (phagocyte), CD68-BV510 (pan-macrophage) from AbD serotec and CCR7-AF647 (M1 macrophage), CD206-FITC (M2a macrophage) from Bioss and CD163-PE (M2c macrophage) from LSBio. Single cell suspensions were first stained for the surface markers CD45, CD11b, CD163, CD206, CCR7 (macrophage panel), or CD45, CD3, CD4, CD25, CD8a (T cell panel). Cells were then stained for intracellular staining (CD68) as previously described^16^. Data were acquired using Gallios flow cytometer (Beckman Coulter) and fluorescence minus one (FMO) samples were used to set the gates. Evaluation was performed using FlowJo v10 software.

### Histology

The right Achilles tendon callus and calf muscles were harvested and kept in 4% phosphate buffered formaldehyde. To keep the samples straight and oriented, each sample was placed in a small petri dish that was half covered with dried silicon gel on the bottom and then samples were kept straight during fixation by passing a needle through the proximal and distal parts of the harvested tissue. The needles were fixed in the silicone gel. This was followed by dehydration steps and embedding in paraffin. The samples were sectioned (4 µm thickness) parallel to the longitudinal axis in the frontal plane of the tendon until the whole tendon was visualized in one section. One glass-slide containing two selected sections were prepared for each animal. Sections were stained with picrosirius red. The sections were made by a blinded technician. Two samples were excluded from blinded evaluation due to low technical quality (one dexamethasone, one control).

An experienced blinded investigator (PA) graded the samples for degree of collagen orientation and organization as either low, middle, or high. The scoring was repeated by the same blinded investigator after 16 weeks. Later, another blinded investigator with less experience of microscopy (MH) scored the specimens, with instruction to estimate the degree of collagen fiber alignment with the direction of tendon traction. In case of a crimp patterns, this should be regarded as a sign of high organization. Inter- and intrarater correlations are reported under results.

### Statistics

The hypothesis was that inflammation disturbs the remodeling phase of tendon healing. The specific hypothesis was that dexamethasone given days 5–9 leads to an increased force at failure on mechanical testing. This was tested with Student’s t-test. Normality was confirmed with Shapiro-Wilk’s test.

When we had seen the mechanical results, i.e. increased strength after late dexamethasone treatment, we formulated two secondary hypotheses, namely that this treatment would also improve tissue organization and change the composition of leukocyte subpopulations in the healing tissue. The histological score (graded 1, 2, 3) and the proportion of CD8a T cells were tested with Mann-Whitney’s U test.

## Electronic supplementary material

Supplementary information
