# Supplementary material for: Systemic corticosteroids improve tendon healing when given after the early inflammatory phase
Source: Sci Rep. 2017 Sep 29;7:12468. doi: 10.1038/s41598-017-12657-0 (PMC5622078; doi:10.1038/s41598-017-12657-0)
Supplement: Supplementary file 1 — Supplementary information [file 41598_2017_12657_MOESM1_ESM.pdf]

Supplementary Information

Systemic corticosteroids improve tendon healing when given after the early inflammatory phase

Parmis Blomgran[1], Malin Hammerman[1], Per Aspenberg[1]

[1] Department of Clinical and Experimental Medicine, Linkoping University, Sweden

Corresponding Author: parmis.blomgran@gmail.com

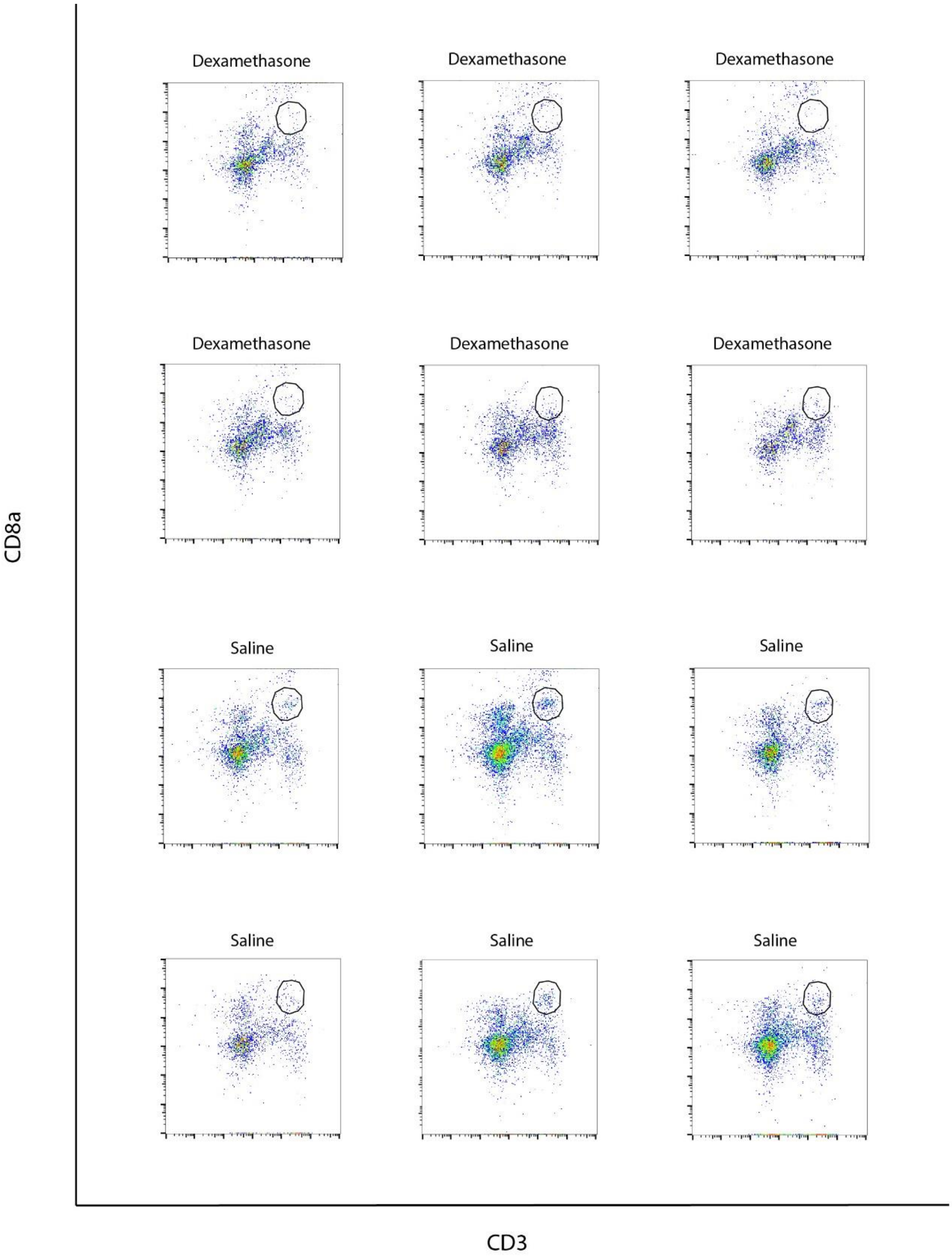

Supplementary Figure S1. Flow plots showing a distinct $CD3^{+}CD8a^{+}$ population in the saline group and few or non-existing $CD3^{+}CD8a^{+}$cells in the dexamethasone treated group 12 days after surgery. N=6 animals in each group.
